# Effect of chemoradiotherapy on the dynamics of circulating lymphocyte subsets in patients with non-metastatic nasopharyngeal carcinoma

**DOI:** 10.3389/fonc.2025.1521836

**Published:** 2025-02-12

**Authors:** Lilan Yi, Yinfang Gu, Longhua Guo, Xiaofang Zou, Guowu Wu

**Affiliations:** ^1^ Department of Oncology, Cancer Center, Meizhou People’s Hospital (Huangtang Hospital), Meizhou Academy of Medical Sciences, Meizhou, Guangdong, China; ^2^ Guangdong Engineering Technological Research Center of Clinical Molecular Diagnosis and Antibody Drugs, Meizhou, Guangdong, China

**Keywords:** nasopharyngeal carcinoma, lymphocyte subsets, chemoradiotherapy, dynamics, immune function

## Abstract

**Background:**

Chemoradiotherapy (CRT) is the primary and most effective treatment for non-metastatic nasopharyngeal carcinoma (NPC), exerting antitumor effects by modulating immune cells. Distinct subpopulations of immune cells exhibit specific sensitivity to CRT. This study aimed to characterize the dynamics of the proportions and absolute counts of peripheral circulating lymphocyte subsets in non-metastatic NPC before and after CRT, and to elucidate their association with clinical responses.

**Methods:**

A total of 91 patients with non-metastatic NPC were enrolled. Flow cytometry was employed to detect the expression of CD3, CD4, CD8, CD56, and CD19 on peripheral blood cells. The composition of lymphocyte subsets before treatment, post-completion of CRT, and one month following CRT was retrospectively analyzed. Further, the relationship between the composition of circulating lymphocyte subpopulations and distinguish clinical responses was evaluated.

**Results:**

The proportion of CD3^+^ T cells showed an initial increase followed by a significant decrease at baseline, post-completion of CRT, and one month following CRT. The proportions of CD3^+^CD4^+^ T cells, CD4^+^/CD8^+^ ratio, and CD19^+^ B cells continued to decline at baseline, post-completion of CRT, and one month following CRT, while the proportions of CD3^+^CD8^+^ T cells and CD16^+^CD56^+^ NK cells progressively increased. The absolute counts of circulating lymphocyte subsets, including CD3^+^ T cells, CD3^+^CD4^+^ T cells, CD3^+^CD8^+^ T cells, CD45^+^, CD19^+^ B cells, and CD16^+^CD56^+^ NK cells, demonstrated a trend of initial decrease followed by an increase at baseline, post-completion of CRT, and one month following CRT. Patients with complete response (CR) and partial response (PR) presented similar dynamic trends in the percentages and absolute counts of circulating lymphocyte subpopulations at baseline, post-completion of CRT, and one month following CRT. The proportions and absolute counts of CD3^+^CD4^+^ T cells in CR patients were distinctly higher than those in PR patients at the end of CRT, whereas the absolute counts of CD16^+^CD56^+^ NK cells were remarkably lower in CR patients compared to PR patients. The baseline proportion and absolute count of CD19^+^ B cells, as well as the absolute count of CD3^+^CD4^+^ T cells, were significantly higher in CR patients compared with PR patients.

**Conclusion:**

CRT induced dynamic alterations in the peripheral lymphocyte profile of non-metastatic NPC patients. Assessing the variations in the distribution of circulating lymphocyte subsets among patients with different clinical treatment responses will be helpful in developing protocols for the concurrent utilization of immunotherapeutic drugs and CRT.

## Introduction

Nasopharyngeal carcinoma (NPC) is a tumor originating from the nasopharyngeal mucosa and is a common malignancy of the head and neck in China. According to the GLOBOCAN 2022 report, there were 120,416 newly diagnosed cases of NPC globally in 2022, resulting in 73,476 associated fatalities (1). These statistics underscore the significant impact of NPC, especially in areas where the disease is prevalent. NPC is highly responsive to radiotherapy, and radical radiotherapy alone is the preferred treatment for early-stage patients. However, the efficacy of radiotherapy alone is not optimal for patients with locally advanced NPC, which has led to the clinical adoption of combined chemoradiotherapy (CRT) approaches. In a recent study, Luo et al. posited that NPC should be conceptualized as an ecological disease, highlighting the dynamic interplay between cancer cells and the tumor microenvironment (2). The research elucidated cancer progression through an ecological lens, suggesting that cancer cells can be likened to “invasive species,” with their metastatic processes characterized by multidirectional dissemination within the ecosystem. Regulation of immune cell homeostasis induced by treatment is critically paramount, as it may interfere with antitumor immune responses (3). CRT could trigger significant alterations in immune-related parameters, such as the composition, phenotype, and function of immune effector cells (4). Thus, monitoring the abundance or activation state of these markers during treatment may provide valuable tools for predicting clinical outcomes (5).

The occurrence, development, and outcome of malignant tumors are intricately linked to the immune function of the human body. The response of the immune system affects cancer prognosis; thus, immune status may serve as a valuable predictive biomarker (6–8). Compared to tumor-infiltrating lymphocytes, circulating lymphocyte subsets in peripheral blood are easier to detect and obtain, which exhibit reproducible measurements. Clinical information on circulating lymphocyte subsets, encompassing levels of T cells, B cells, and NK cells, is frequently employed as hematological markers of immune function (9, 10). T lymphocytes primarily mediate cellular immunity, recognize, label, and clear tumor cells, and play a crucial role in tumor resistance. T cells, which are integral to the adaptive immune system, are predominantly classified into CD3^+^CD4^+^ T cells and CD3^+^CD8^+^ T cells according to their surface differentiation antigens. CD19 serves as a crucial antigen and marker on the surface of B cells, whereas two principal surface antigens, CD16 and CD56, are utilized for the identification and classification of natural killer (NK) cell subsets (11–13). Different subsets of immune cells exhibit specific sensitivities to CRT. Prior investigations have indicated an association between certain levels of circulating lymphocyte subsets and a poor prognosis in NPC patients (14–17). Nevertheless, the dynamic changes in circulating lymphocyte subsets before and after CRT and their associations with clinical treatment response of NPC patients remain unclear.

Accordingly, the composition and dynamics of major lymphocyte subsets in the peripheral blood of non-metastatic NPC patients were retrospectively analyzed at three distinct time points: before treatment, at the end of CRT, and one month after CRT. Additionally, the association between the dynamic alterations in circulating lymphocyte subpopulations and the clinical responses to treatment was elucidated.

## Materials and methods

### Study population

The study population comprised of patients with NPC who were initially diagnosed and underwent CRT at Meizhou People’s Hospital from January 2018 to March 2021. The determination of lymphocyte subpopulations was a routine examination conducted on a voluntary basis, as informed by the doctors for nearly all lymphoma and other tumor patients. This study focused primarily on the peripheral immune profiles in patients with non-metastatic NPC. Immune data was collected retrospectively at three different time points: prior to any treatment, at the end of CRT, and one month after CRT. The inclusion criteria for this study were as follows: (1) newly diagnosed, pathologically confirmed nasopharyngeal undifferentiated non-keratinizing carcinoma; (2) age 18-75 years, with no distant metastasis; (3) receiving CRT (induction chemotherapy followed by concurrent chemoradiotherapy; concurrent chemoradiotherapy; induction chemotherapy followed by radical radiotherapy); (4) complete TBNK (T cells, B cells, NK cells) subsets and follow-up data; (5) no local radiotherapy boost for residual lesions; (6) ECOG (Eastern Cooperative Oncology Group) performance status score of 0-2; (7) normal heart, kidney, and liver functions. Exclusion criteria were as follows: (1) presence of other types of malignancies; (2) receiving hormone therapy or other treatments (targeted therapy or surgery); (3) presence of additional chronic diseases or known/suspected active autoimmune diseases. This study was approved by the Medical Ethics Committee of the Meizhou People’s Hospital (Huangtang Hospital) (the ethical approval number: 2024-C-43). The Committee waived the requirement for informed consent as it was a retrospective study. All data were collected in accordance with approved guidelines.

### Clinicopathological variables and circulating lymphocyte phenotype detection

The collected clinicopathological data included age at diagnosis, gender, smoking history, tumor TNM stage, induction chemotherapy regimen, concurrent chemotherapy regimen, use of nimotuzumab, radiotherapy dose, comprehensive treatment plan, and clinical efficacy evaluation. Laboratory data for analysis consisted of the percentage and absolute count of peripheral blood lymphocytes at three time points: pre-treatment, at the end of CRT, and one month after CRT. This encompassed T cells characterized by CD3 expression (CD3^+^ T cells), B cells defined by CD19 expression (CD19^+^ B cells), and T lymphocyte subsets identified by the presence of CD4 (CD3^+^CD4^+^ T cells) and CD8 (CD3^+^CD8^+^ T cells). The combination of CD56 and CD16 facilitated the identification of natural killer (NK) lymphocyte populations. CD45 was used to identify leukocyte antigens in peripheral blood. The clinical efficacy of CRT was evaluated using magnetic resonance imaging (MRI). According to the Response Evaluation Criteria in Solid Tumors (RECIST) version 1.1, clinical efficacy was classified into complete response (CR), partial response (PR), stable disease (SD), and progressive disease (PD).

According to the protocol, the staining steps for lymphocyte subsets were as follows: Peripheral blood lymphocytes were analyzed using six-color flow cytometry (FACSCanto II, BD Biosciences, San Jose, CA, USA). Fifty microliters of EDTA-K2 anticoagulated venous blood were added to the bottom of the absolute counting tube using a reverse pipetting method. Then, 20 uL of fluorescein-labeled monoclonal antibodies (6C-TBNK Lymphocyte Test Kit, BD Biosciences, San Jose, CA, USA) were added to the bottom of the absolute counting tube. The mixture was gently vortexed and incubated in the dark for 15 minutes for staining. Next, 450 uL of lysing solution was added to the tube, gently vortexed, and allowed to react for 15 minutes before the samples were analyzed using flow cytometry. All instruments and reagents were purchased from BD Biosciences (USA). In accordance with the flow cytometry platform of our hospital, the normal reference ranges were defined as follows: CD3^+^ T cells (48.0–82.0%), CD4^+^ CD8^+^ T cells (13.0–42.0%), CD3^+^ CD4^+^ T cells (21.0–46.0%), CD4/CD8 (0.71-2.78), CD19^+^ B cells (4.0–18.0%), CD16^+^CD56^+^ NK cells (7.0–41.0%), CD3^+^ T cells absolute count (668-2474/uL), CD3^+^CD8^+^ T cells absolute count (161-1159/uL), CD3^+^CD4^+^ T cells absolute count (292-1309/uL), CD19^+^ B cells absolute count (79-562/uL), CD16^+^CD56^+^ NK cells absolute count (142-1270/uL).

### Statistical analysis

All statistical data analyses in this study were conducted using R software Version 4.2.1 (https://www.R-project.org). Descriptive statistics were employed to assess the baseline characteristics of patients. Results were presented as the median of the interquartile range for the percentage or absolute count of positive lymphocytes. Differences in the percentages or absolute counts of immune cells pre- and post-CRT were detected using the Wilcoxon test or the Kruskal-Wallis test for related samples. The alteration in the proportion of circulating lymphocytes pre- and post-CRT was defined as Δ lymphocyte = post-CRT lymphocyte proportion/baseline lymphocyte proportion. The relationship between Δ lymphocyte and clinical characteristics was estimated using the Wilcoxon rank sum test. A P-value of less than 0.05 was considered statistically significant.

## Results

### Patient characteristics

A total of 91 patients met the inclusion criteria and were comprehensively evaluated. All patients completed CRT. Among the 91 non-metastatic NPC patients, 73 (80.22%) were male, and 18 (19.78%) had a history of smoking. The median age of the patients was 49 years (range 42-54 years). According to the 8th edition of the AJCC staging system, there were 70 patients with stage IVA, 19 with stage III, and 2 with stage II. The primary induction chemotherapy regimen consisted of docetaxel combined with platinum, which was used in 85 patients (93.41%), followed by paclitaxel combined with platinum in 4 patients (4.39%). There were three treatment modalities: 85 patients (93.41%) received induction chemotherapy followed by concurrent CRT, and 5 patients (5.49%) received induction chemotherapy followed by radical radiotherapy. The main concurrent chemotherapy drug used during radiotherapy was cisplatin, which was administered to 68 patients (74.73%). Nimotuzumab was utilized in 76 patients (83.52%) during radiotherapy. All patients underwent intensity-modulated radiotherapy (IMRT). The doses were as follows: primary tumor (PGTV) received 70-74 Gy/30-33 fractions, metastatic lymph nodes (PGTVnd) received 70 Gy/30-33 fractions, subclinical lesions and high-risk areas (PCTV1) received 60 Gy/30-33 fractions, and low-risk areas (PCTV2) received 50-52 Gy/28 fractions. At the end of CRT, 26 patients (28.57%) achieved CR. The baseline characteristics of patients are shown in **Table 1**.

**Table 1:** Clinical characteristics of 91 patients with non-metastatic nasopharyngeal carcinoma.

Characteristic	N (%) or median range	Characteristic	N (%) or median range
**Gender**		**Induction chemotherapy regimen**	
Male	73 (80.22)	DP	85 (93.41)
Female	18 (19.78)	TP	4 (4.39)
**Age (years)**		Others	2 (2.20)
median (range)	49 (42-54)	**Treatment method**	
**Smoking history**		IC + RT	5 (5.49)
Non-smoker	73 (80.22)	IC+CCRT	85 (93.41)
Smoker	18 (19.78)	CCRT	1 (1.10)
**T stage**		**Chemotherapy regimen of CCRT**	
T1	13 (14.29)	Cisplatin	68 (74.73)
T2	15 (16.48)	Carboplatin	3 (3.29)
T3	29 (31.87)	Nedaplatin	13 (14.29)
T4	34 (37.36)	Lobaplatin	2 (2.20)
**N stage**		None	5 (5.49)
N0	2 (2.20)	**Nimotuzumab**	
N1	20 (21.98)	Yes	76 (83.52)
N2	24 (26.37)	No	15 (16.48)
N3	45 (49.45)	**Total dose of RT (Gy)**	
**TNM stage**		Median (range)	74 (73.92-74)
II	2 (2.20)	**CRT efficiency**	
III	19 (20.88)	CR	26 (28.57)
IVA	70 (76.92)	PR	65 (71.43)

DP, Docetaxel plus Platinum; TP, Paclitaxel plus Platinum; IC, Induction chemotherapy; RT, Radiotherapy; CCRT, Concurrent chemoradiotherapy; CRT, Chemoradiotherapy; CR, Complete response; PR, Partial response. Bold values means Clinical characteristics.

### Dynamics of circulating lymphocyte subsets after chemoradiotherapy

**Figure 1:**
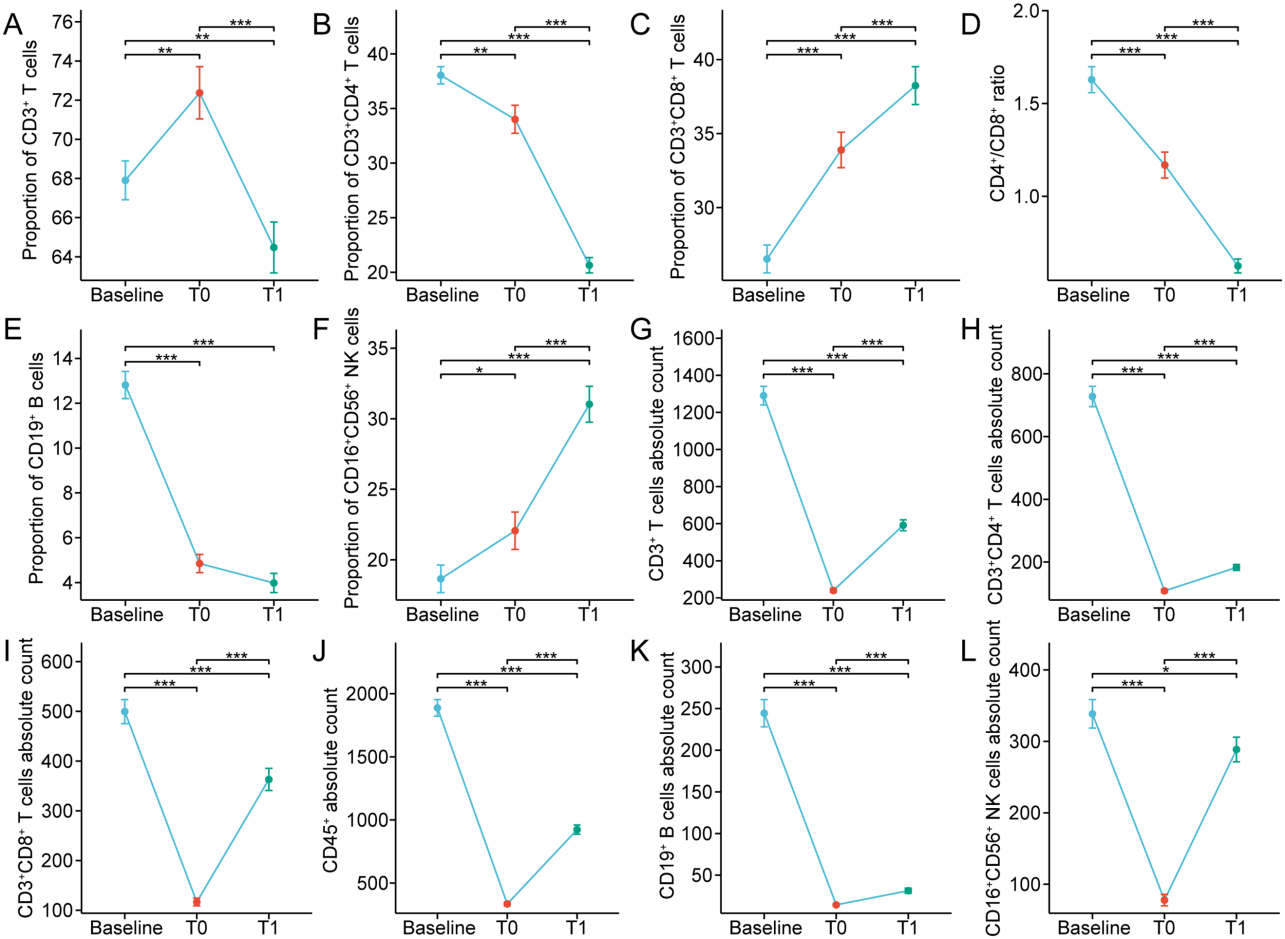
**(A-F)** Percentages and **(G-L)** absolute counts of lymphocyte subpopulations in peripheral blood at baseline and after chemoradiotherapy in non-metastatic nasopharyngeal carcinoma patients. T0 represented the values at the end of chemoradiotherapy; T1 represented the values at one month after chemoradiotherapy. *p < 0.05; **p < 0.01; ***p < 0.001.

The expression of CD3, CD4, CD8, CD56, and CD19 on peripheral blood cells was detected by flow cytometry and the composition of lymphocyte subsets before treatment, post-completion of CRT, and one month following CRT was retrospectively analyzed (**Supplementary Figure S1**). **Figure 1** and **Table 2** depicted the variations in circulating lymphocyte subsets before treatment, post-completion of CRT, and one month following CRT. In comparison to the baseline, the proportion of CD3^+^ T cells exhibited a significant increase at the end of CRT (p<0.01), followed by a marked decrease one month later (p<0.01) (**Figure 1A**). The proportion of CD3^+^CD4^+^ T cells, the CD4^+^/CD8^+^ ratio, and the proportion of CD19^+^ B cells continued to decline at baseline, at the end of CRT, and one month after CRT (p<0.001) (**Figures 1B, D, E**); whereas, the proportions of CD3^+^CD8^+^ T cells and CD16^+^CD56^+^ NK cells continued to rise (p<0.001) (**Figures 1C, F**). The absolute counts of circulating lymphocyte subsets demonstrated a trend of initial decrease followed by an increase at baseline, at the end of CRT, and one month after CRT. Compared to baseline, the absolute counts of CD3^+^ T cells, CD3^+^CD4^+^ T cells, CD3^+^CD8^+^ T cells, CD45^+^, CD19^+^ B cells, and CD16^+^CD56^+^ NK cells sharply decreased at the end of CRT (p<0.001) but rapidly increased one month after CRT (p<0.001) (**Figures 1G-L**).

**Table 2:** Chemoradiotherapy-induced alterations of circulating lymphocyte subpopulation.

Variables	Baseline	T0	T1	Normal reference range
Proportion of CD3^+^ T cells	68.98 (63.15 - 74.42)	73.59 (66.68 - 82.08)	65.27 (56.62 - 72.76)	48-82%
Proportion of CD3^+^CD8^+^ T cells	26.34 (19.955 - 30.545)	31.40 (26.67 - 41.03)	37.04 (29.38 - 47.055)	13-42%
Proportion of CD3^+^CD4^+^ T cells	36.89 (32.855 - 44.775)	33.22 (24.795 - 42.115)	19.85 (15.89 - 24.275)	21-46%
Proportion of CD19^+^ B cells	12.21 (8.59 - 16.085)	3.67 (2.26 - 5.875)	2.82 (1.76 - 4.18)	4-18%
Proportion of CD16^+^CD56^+^ NK cells	16.89 (11.675 - 22.665)	19.19 (12.585 - 29.395)	29.13 (23.885 - 37.935)	7-41%
CD4^+^/CD8^+^ ratio	1.61 (1.165 - 2.09)	1.05 (0.65 - 1.615)	0.53 (0.375 - 0.825)	0.71-2.78
CD3^+^ T cells absolute count	1270 (934 - 1619)	221 (164 - 308.5)	531 (385 - 697.5)	668-2474/uL
CD3^+^CD8^+^ T cells absolute count	471 (346 - 630)	99 (65.5 - 134.5)	312 (213.5 - 458)	161-1159/uL
CD3^+^CD4^+^ T cells absolute count	657 (526 - 919.5)	99 (71 - 128.5)	158 (129.5 - 212)	292-1309/uL
CD19^+^ B cells absolute count	198 (126.5 - 336)	11 (8 - 18)	24 (16 - 37)	79-562/uL
CD16^+^CD56^+^ NK cells absolute count	300 (207.5 - 438.5)	56 (35.5 - 98.5)	232 (171.5 - 382)	142-1270/uL
CD45^+^ absolute count	1890 (1429 - 2272.5)	315 (236 - 412.5)	873 (637 - 1153)	–

CRT, chemoradiotherapy; T0 represented the values at the end of chemoradiotherapy; T1 represented the values at one month after chemoradiotherapy.

Furthermore, the median percentages of CD3^+^ T cells, CD3^+^CD8^+^ T cells, and CD16^+^CD56^+^ NK cells were elevated or declined within the normal ranges before and after CRT (**Table 2**). At baseline, the proportion of CD19^+^ B cells in 89/91 (97.80%) patients was within normal range, but at the end of CRT and one month after CRT, the percentages of CD19^+^ B cells of most patients (50/91, 54.95%; 67/91, 73.63%) were decreased, with the median further declining. The median percentage of CD3^+^CD4^+^ T cells was normal at both baseline and the end of CRT, but, one month after CRT, the proportion of CD3^+^CD4^+^ T cells in 52/91 (57.14%) patients was lower than the normal range. Similarly, we observed that the CD4^+^/CD8^+^ ratio in 60/91 (65.93%) patients was lower than normal one month after CRT. At baseline, the absolute counts of CD3^+^ T cells in 82/91 (90.11%) patients were normal; whereas, the absolute counts of CD3^+^ T cells of most patients (91/91, 100%; 65/91, 71.43%) were decreased at the end of CRT and one month after CRT. The median absolute count of CD3^+^CD8^+^ T cells was within the reference range at baseline and one month after CRT, but at the end of CRT, the CD3^+^CD8^+^ T cells absolute count was declined in 72/91 patients (79.12%). The median absolute count of CD3^+^CD4^+^ T cells was within the normal range at baseline, but at the end of CRT and one month after CRT, the CD3^+^CD4^+^ T cells absolute counts were decreased in most patients (91/91, 100%; 84/91, 92.31%). Similarly, at the end of CRT and one month after CRT, the CD19^+^ B cells absolute counts were decreased in 91/91 (100%) and 86/91 patients (94.51%), respectively. At the end of CRT, the CD16^+^CD56^+^ NK cells absolute counts were declined in 80/91 (87.91%) patients, whereas the median count was normal at baseline and one month after CRT. These results indicate that CRT triggers complex changes in the composition of circulating lymphocytes in the peripheral blood of non-metastatic NPC.

**Figure 2:**
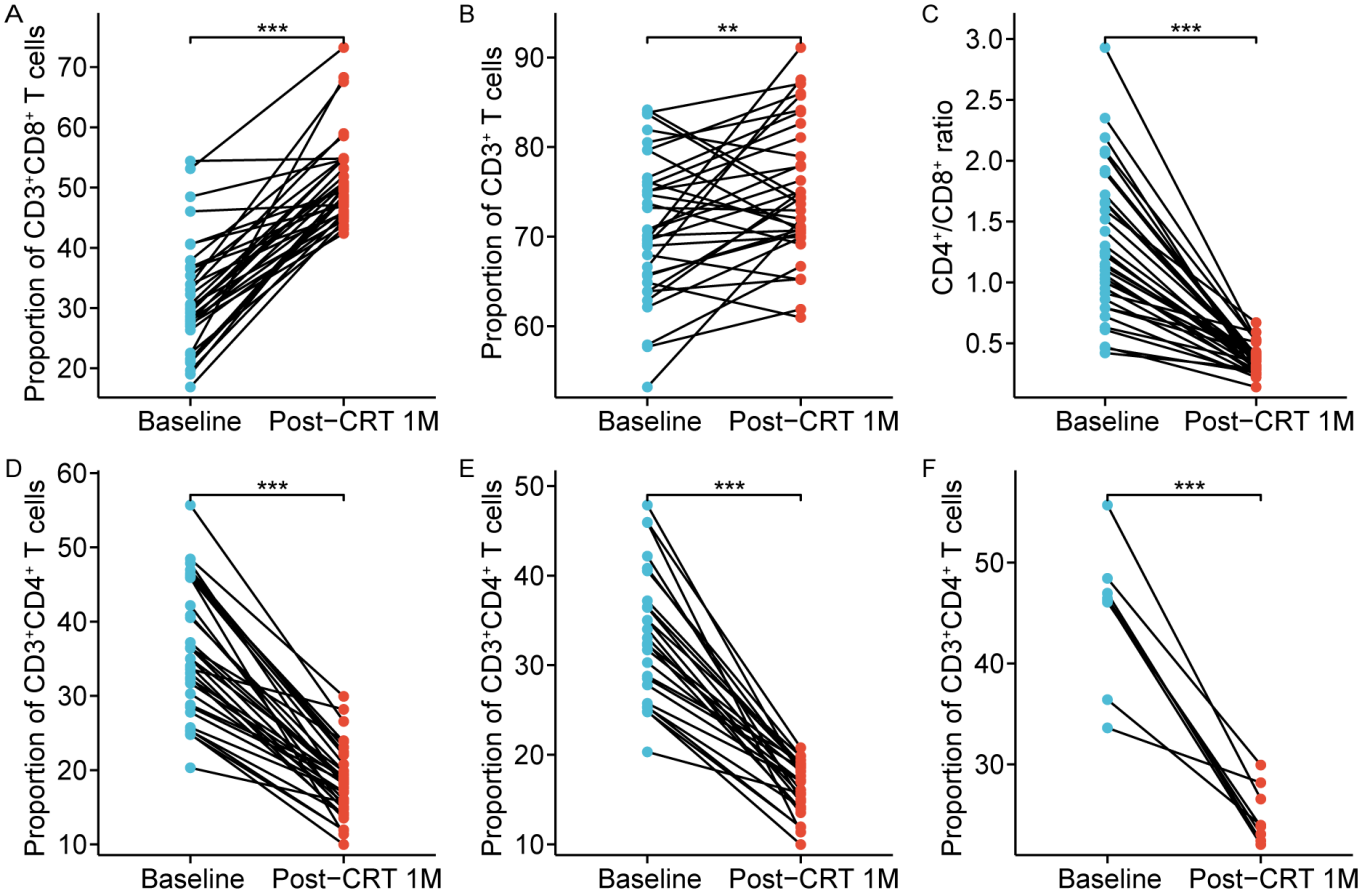
Chemoradiotherapy-induced alterations in 34 patients with circulating high CD8^+^ T cells. The proportion of circulating CD8^+^ T cells **(A)**, CD3^+^ T cells **(B)**, CD4^+^/CD8^+^ ratio **(C)**, and CD4^+^ T cells **(D)** after chemoradiotherapy. **(E)** The proportion of CD4^+^ T cells were low in 26 cases (normal range, 21.0–46.0%). **(F)** The proportion of CD4^+^ T cells were normal in 8 cases (normal range, 21.0–46.0%). Post-CRT 1M represented the values at one month after chemoradiotherapy. **p < 0.01; ***p < 0.001.

Notably, CD8^+^ T cells primarily differentiate into cytotoxic T cells, which directly eradicate tumor cells. Thus, we further focused on the alterations in CD8^+^ T cells after CRT. The proportions of CD8^+^ T cells in 34/91 (37.36%) patients after CRT were higher than 42% (normal reference range, 13.0-42.0%), but at baseline, the proportions of CD8^+^ T cells in only 5/91 (5.49%) patients was over 42% (**Figure 2A**). Also, we found that in these 34 patients, the proportions of CD3^+^ T cells were all less than 48% (normal reference range, 48.0-82.0%) after CRT, and the CD4^+^/CD8^+^ ratio was lower than 0.71 (normal reference range, 0.71-2.78) (**Figures 2B-C**). It is well-known that a low CD4^+^/CD8^+^ ratio can result from a diminished proportion of CD4^+^ T cells and/or an augmented proportion of CD8^+^ T cells. After CRT, among the 45 patients with elevated proportion of CD8^+^ T cells, the proportions of CD4^+^ T cells in 26 patients were less than 21%, and the proportions of CD4^+^ T cells in 8 patients were over 46% (normal reference range, 21.0-46.0%) (**Figures 2D-F**). These results reveal that the overall immune function in non-metastatic NPC remains normal after CRT, and the increased percentages of CD8^+^ T cells might be attributed to the immune system response in generating more cytotoxic T cells following CRT.

### Correlation between peripheral lymphocyte subsets and clinical characteristics

**Figure 3:**
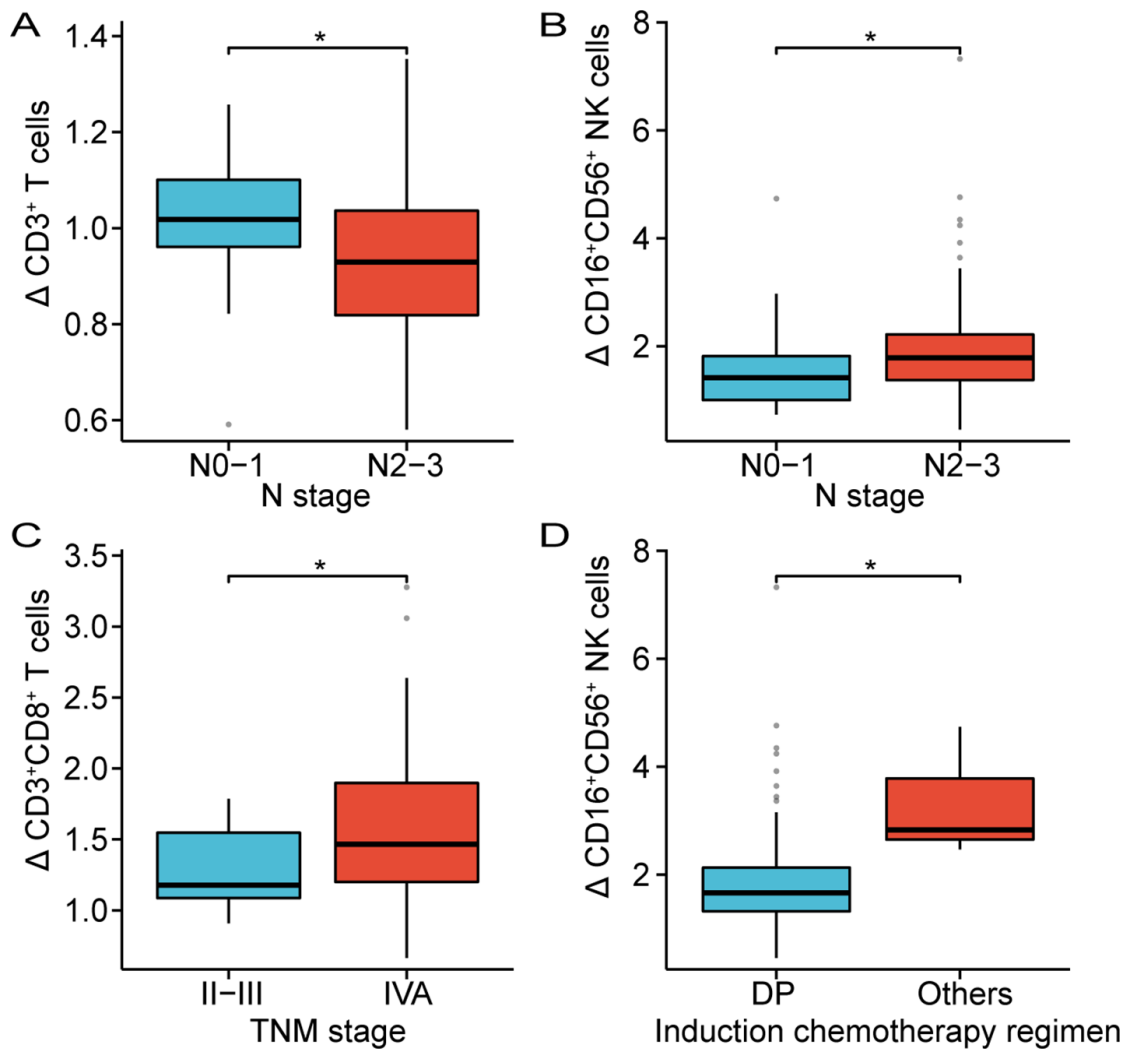
Correlation between the alterations of CD3^+^ T cells **(A)**, CD8^+^ T cells **(C)** and CD16^+^CD56^+^ NK cells **(B, D)** and clinical parameters. DP, Docetaxel plus Platinum; *p < 0.05.

The clinical characteristics and treatment modalities of patients may influence the changes in the composition of circulating lymphocyte subsets before and after CRT. As shown in **Table 3**, the results suggested that changes in circulating CD3^+^ T cells and CD16^+^CD56^+^ NK cells were associated with the N stage. Patients with stage N2-3 existed a significantly lower proportion of CD3^+^ T cells after CRT compared to patients with stage N0-1 (N0-1 vs N2-3, p = 0.03). The proportion of CD16^+^CD56^+^ NK cells was closely higher in patients with stage N2-3 after CRT than in those with stage N0-1 (N0-1 vs N2-3, p = 0.02). Patients with stage IVA displayed a dramatically higher proportion of CD3^+^CD8^+^ T cells after CRT compared with patients with stage II-III (II-III vs IVA, p = 0.02). Furthermore, patients who received DP induction chemotherapy had a markedly lower proportion of CD16^+^CD56^+^ NK cells after CRT than in patients who received other induction chemotherapy regimens (DP vs. others, p = 0.02). However, there was no significant correlation between changes in the composition of circulating lymphocyte subsets and factors such as gender, T stage, use of nimotuzumab, concurrent chemotherapy regimen, and treatment modality. **Figure 3** further implicated the association between changes in CD3^+^ T cells, CD16^+^CD56^+^ NK cells, and CD3^+^CD8^+^ T cells with TNM stage and induction chemotherapy regimen.

**Table 3:** Correlation between lymphocyte subsets alterations and clinical characteristics.

Variables	Gender (Male vs Female)		Smoking history (Non-smoker vs Smoker)		T stage (T1-2 vs T3-4)		N stage (N0-1 vs N2-3)		TNM stage (II-III vs IVA)		Induction chemotherapy regimen (DP vs Others)		Nimotuzumab (Yes vs No)		Chemotherapy regimen of CCRT (Cisplatin vs Nedaplatin)		Treatment method (IC+CCRT vs IC + RT)	
	z	p	z	p	z	p	z	p	z	p	z	p	z	p	z	p	z	p
Δ CD3^+^ T cell	0.072	0.942	-0.565	0.573	-0.208	0.835	-2.189	**0.031**	0.070	0.071	-0.876	0.383	0.386	0.700	-0.135	0.892	0.696	0.488
Δ CD3^+^CD8^+^ T cell	-0.063	0.533	0.054	0.664	0.057	0.676	-0.115	0.284	0.221	**0.016**	-0.197	0.246	-0.019	0.885	0.051	0.647	-0.164	0.306
Δ CD3^+^CD4^+^ T cell	1.941	0.055	-0.065	0.089	-0.001	0.976	-0.010	0.721	0.004	0.854	-0.083	0.182	0.044	0.366	-0.022	0.685	0.176	0.217
Δ CD16^+^CD56^+^ NK cell	-0.023	0.908	0.190	0.321	-0.057	0.682	0.406	**0.017**	-0.181	0.260	1.314	**0.020**	-0.198	0.233	-0.009	0.964	-0.122	0.672
Δ CD19^+^ B cell	0.054	0.150	-0.031	0.434	-0.023	0.499	0.007	0.813	-0.028	0.404	-0.027	0.559	0.123	0.122	0.033	0.483	0.127	0.181
Δ CD4^+^/CD8^+^ ratio	0.053	0.211	-0.055	0.200	-0.004	0.921	0.011	0.798	-0.065	0.112	0.086	0.280	0.030	0.497	-0.041	0.443	0.216	0.181

DP, Docetaxel plus Platinum; IC, Induction chemotherapy; RT: Radiotherapy; CCRT, Concurrent chemoradiotherapy. Bold values means P<0.05.

### Association between lymphocyte parameters and clinical response

**Figure 4:**
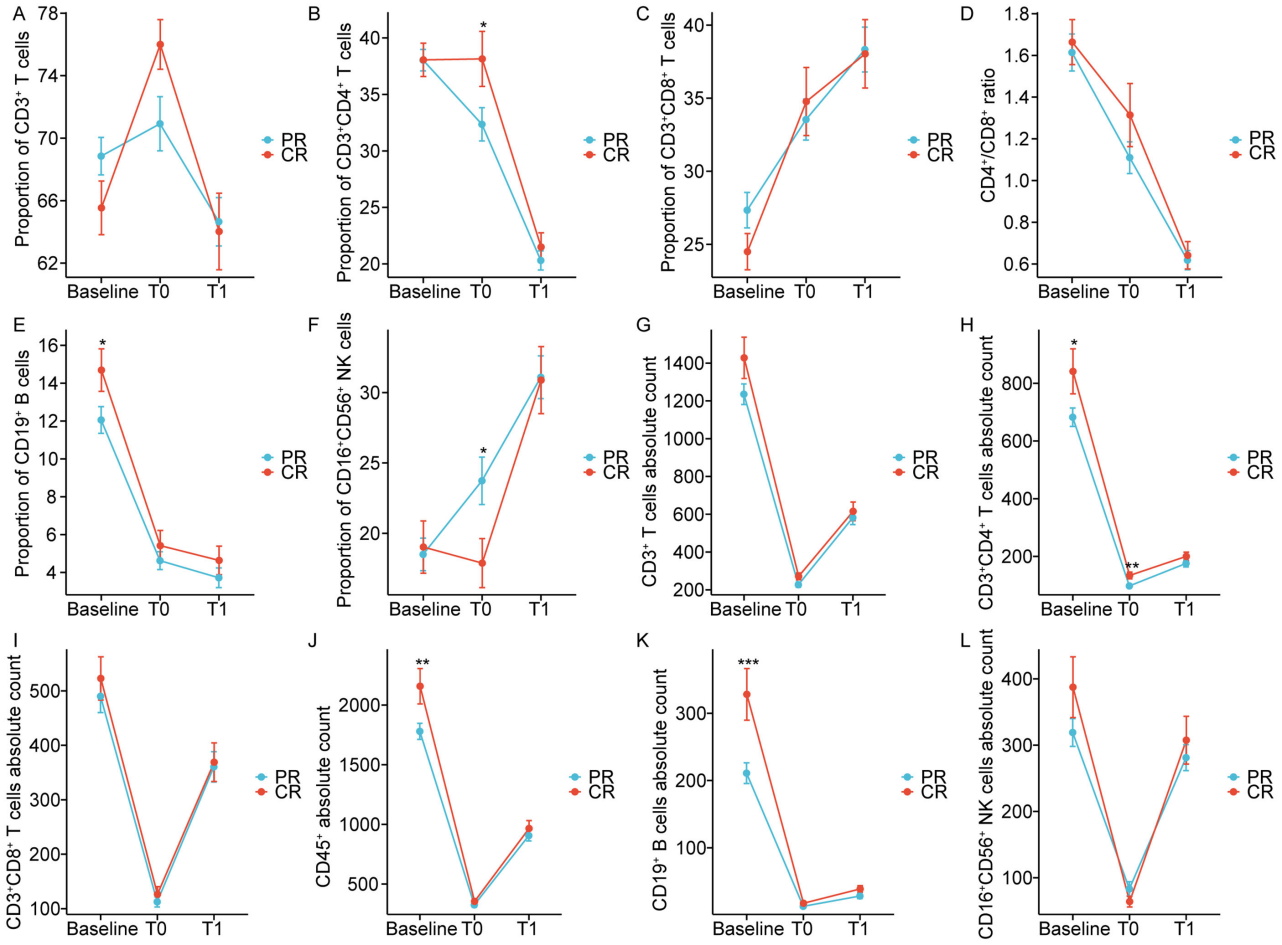
Chemoradiotherapy-induced dynamics of the proportion **(A-F)** and absolute counts **(G-L)** of peripheral lymphocyte subsets based on clinical response. CR: Complete response; PR: Partial response; T0 represented the values at the end of chemoradiotherapy; T1 represented the values at one month after chemoradiotherapy. *p < 0.05; **p < 0.01; ***p < 0.001.

**Figure 5:**
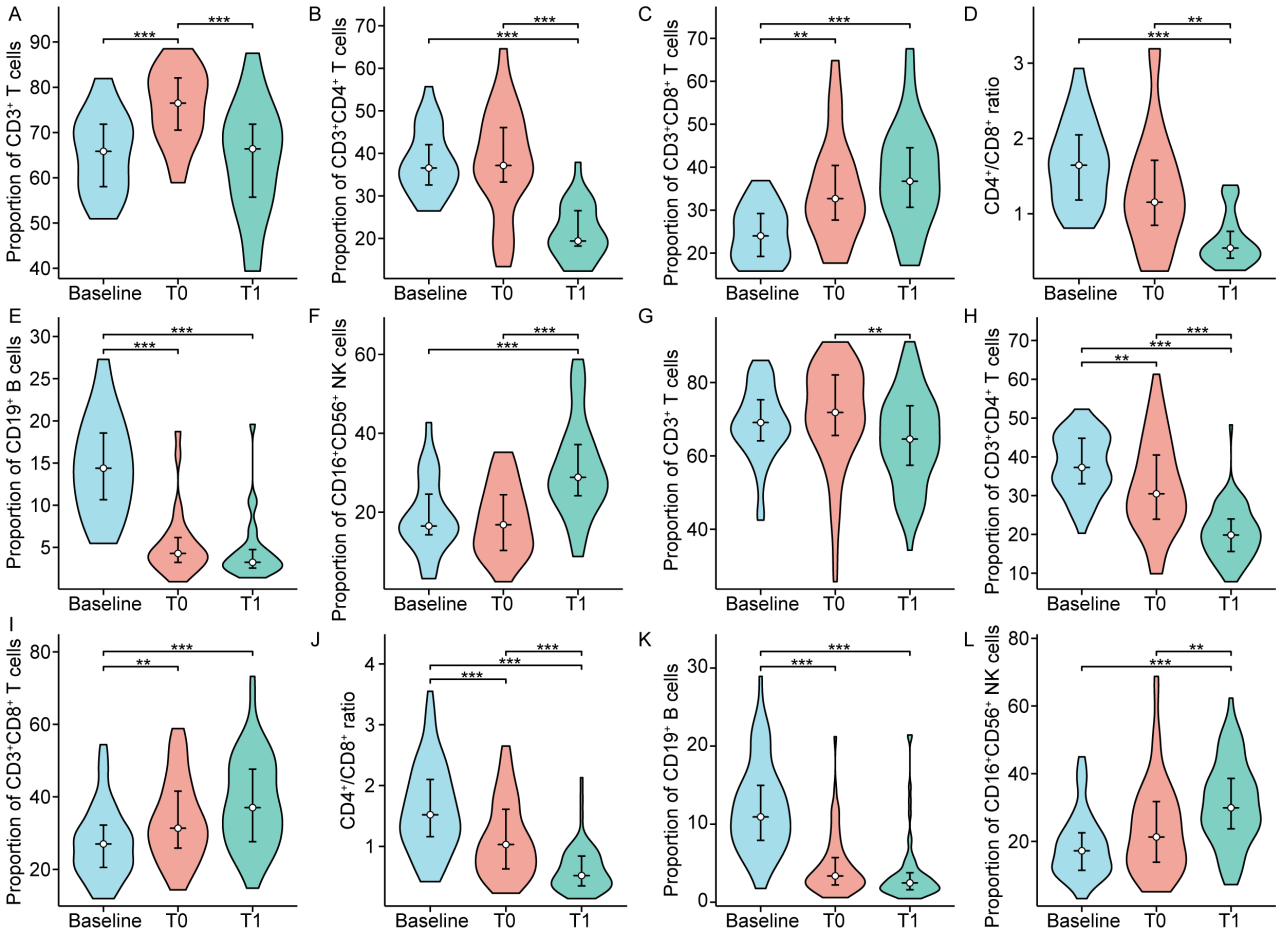
The proportion of peripheral lymphocyte subsets in patients with complete response **(A-F)** and partial response **(G-L)**. T0 represented the values at the end of chemoradiotherapy; T1 represented the values at one month after chemoradiotherapy. **p < 0.01; ***p < 0.001.

**Figure 6:**
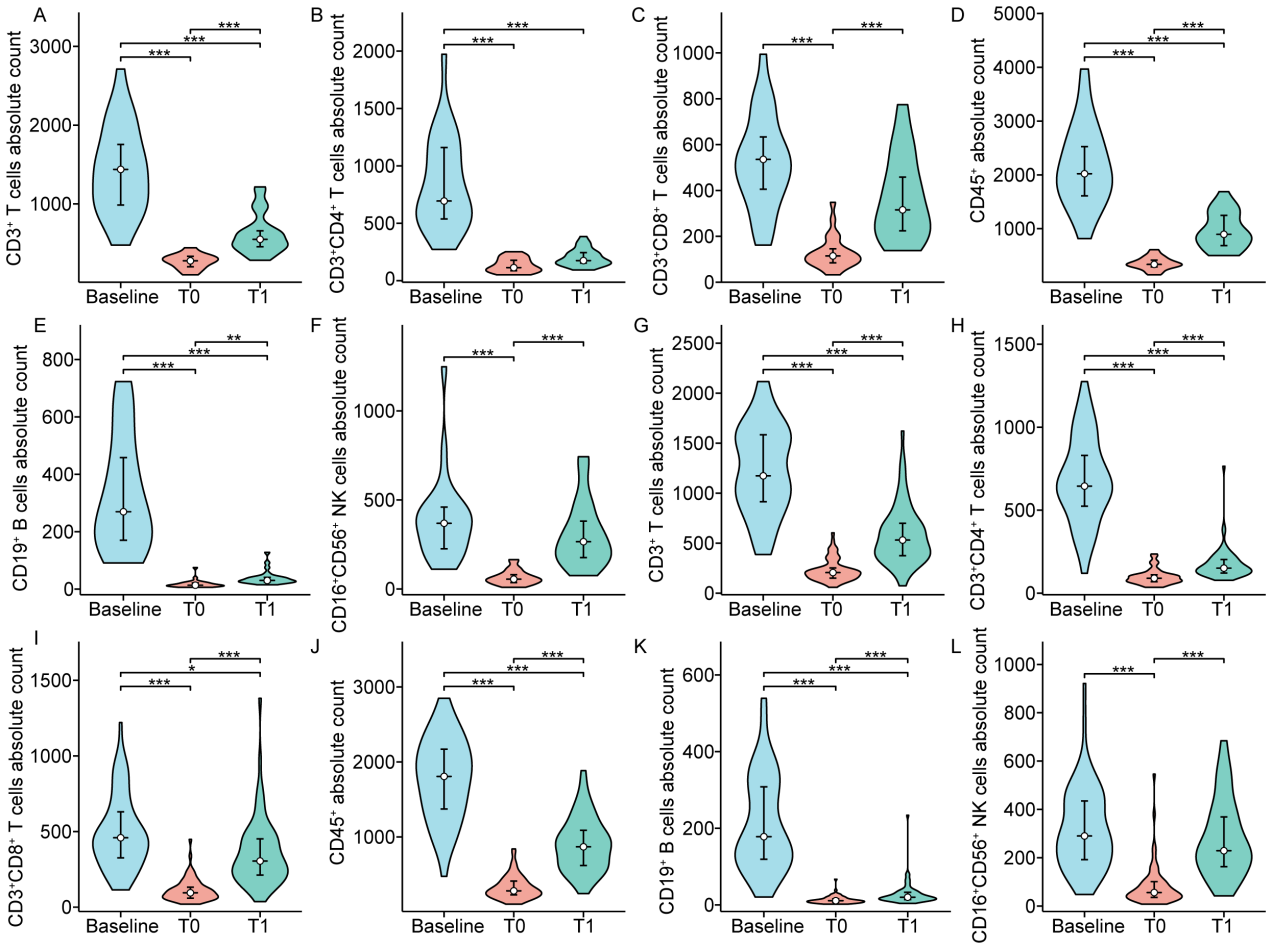
The absolute counts of circulating lymphocyte subpopulation in patients with complete response **(A-F)** and partial response **(G-L)**. T0 represented the values at the end of chemoradiotherapy; T1 represented the values at one month after chemoradiotherapy. *p < 0.05; **p < 0.01; ***p < 0.001.

The impact of dynamically measured immune characteristics on the clinical response was further evaluated in patients with non-metastatic NPC. As shown in **Figure 4**, the dynamic trends of circulating lymphocyte subsets at baseline, at the end of CRT, and one month after CRT were largely consistent between the CR and PR groups, but there were significant differences at specific time points. **Figures 5**, **6** separately illustrated the dynamic changes in the percentages and absolute counts of circulating lymphocyte subsets in the CR and PR groups at baseline, at the end of CRT, and one month after CRT. At the end of CRT, the proportion of CD3^+^CD4^+^ T cells was distinctly higher in the CR group than that in the PR group (p<0.05, **Figure 4B**). The baseline proportion of CD19^+^ B cells was also significantly higher in the CR group (p<0.05, **Figure 4E**). Conversely, at the end of CRT, the percentages of CD16^+^CD56^+^ NK cells were markedly lower in the CR group compared to the PR group (p<0.05, **Figure 4F**). Furthermore, the baseline absolute counts of CD3^+^CD4^+^ T cells, CD45^+^, and CD19^+^ B cells were tightly higher in the CR group than that in the PR group (p<0.05, **Figure 4H**; p<0.01, **Figure 4J**; p<0.001, **Figure 4K**). At the end of CRT, the absolute count of CD3^+^CD4^+^ T cells remained remarkedly higher in the CR group than that in the PR group (p<0.01, **Figure 4H**). Notably, there were no statistical differences in the percentages and absolute counts of lymphocyte subsets between the CR and PR groups one month after CRT. These results ascertained that the dynamic changes of circulating lymphocyte subsets between the CR and PR groups were profoundly impressive, particularly at the end of CRT.

## Discussion

This study concentrated on the dynamic alterations in the composition of circulating lymphocyte subpopulations pre- and post-CRT, and assessed their potential role in the clinical response of patients with non-metastatic NPC. The results indicated that the proportion of CD3^+^ T cells initially increased, followed by a rapid decline at baseline, at the end of CRT, and one month after CRT. The proportions of CD3^+^CD4^+^ T cells and CD19^+^ B cells, as well as the CD4^+^/CD8^+^ ratio, exhibited a continued decline at these time points. Conversely, the proportions of CD3^+^CD8^+^ T cells and CD16^+^CD56^+^ NK cells demonstrated a progressive increase. The absolute counts of circulating lymphocyte subsets, encompassing CD3^+^ T cells, CD3^+^CD4^+^ T cells, CD3^+^CD8^+^ T cells, CD45^+^, CD19^+^ B cells, and CD16^+^CD56^+^ NK cells, demonstrated a dynamic pattern characterized by an initial decline followed by an increase at baseline, at the end of CRT, and one month after CRT. Furthermore, patients in the CR and PR groups exhibited similar dynamic trends in both the percentages and absolute counts of circulating lymphocyte subsets at the specified time points. At the end of CRT, there was a notable increase in both the proportion and absolute count of CD3^+^CD4^+^ T cells in CR patients than those in PR patients. Conversely, the absolute count of CD16^+^CD56^+^ NK cells was significantly lower in CR patients in comparison to PR patients. The baseline proportions of CD19^+^ B cells, absolute counts of CD19^+^ B cells, and CD3^+^CD4^+^ T cells were found to be significantly elevated in CR patients compared to PR patients. These data indicate that CRT participates in the dynamic regulation of the peripheral immune cell composition in patients with non-metastatic NPC. The impact of CRT on immunocyte functions in individuals with non-metastatic NPC is significant. Monitoring changes in circulating lymphocyte subsets throughout treatment may offer valuable information on the patient’s immune status and aid in guiding therapeutic interventions.

In addition to its immunosuppressive effects, CRT also demonstrates immunostimulatory effects that enhance antitumor immune responses (18–20). Previous studies have shown a positive correlation between CRT and the activity of proliferative CD8^+^ tumor-infiltrating lymphocytes (21). The findings of our study suggest a gradual elevation in CD8^+^ T lymphocyte levels and a corresponding decline in CD4^+^ T cell levels subsequent to CRT. Emerging clinical evidence suggests that elevated lymphocyte levels are closely linked to favorable prognosis in different types of human cancers (22, 23). Owing to the potent cytotoxic activity of T cells, previous research predominantly focused on tumor-specific CD8^+^ T cells, while CD4^+^ T cells were recognized as helpers for CD8^+^ T cells. Recent studies have uncovered that CD4^+^ T cells are not a homogeneous cell line with a single function, but rather a diverse population with intricate functions (24, 25). The decline in CD4^+^ T cell proportion following CRT may suggest impaired cell-mediated immunity, while the rise in CD8^+^ T cell proportion could be linked to the activation triggered by the release of tumor-associated antigens prompted by CRT. These results suggest that the dynamic changes in circulating lymphocyte subsets in patients with NPC may reflect a reduction in immune function. Hence, it is imperative to dynamically monitor the alterations in lymphocyte subsets of patients throughout the course of treatment, as enhancing immune function via pharmacological intervention has the potential to positively impact patient prognosis.

In alignment with prior research conducted on breast cancer and esophageal squamous cell carcinoma (ESCC) (26, 27), our results indicate a significant decrease in CD19^+^ B cell levels following CRT. This finding aligns with earlier research, suggesting that B lymphocytes are the most sensitive subset in non-hematologic malignancies. In cancer patients, CD19^+^ B lymphocytes play a crucial role in humoral immunity by binding specifically to B-cell activating factor (BAFF) and producing antibodies against tumor-associated antigens. Moreover, B cells can process and present antigens to induce T-cell immune responses and interact with macrophages and the complement system to effectively eliminate tumor cells (28–30). In our research, we found that both the proportion and absolute count of peripheral blood B cells induced by CRT in non-metastatic NPC patients were below normal levels, suggesting a compromised humoral immune response. Notably, NK cells, a subset of cytotoxic lymphocytes, play a crucial role in enhancing both innate and adaptive immunity by inducing cytolysis and releasing various chemokines and cytokines (31). Researchers have observed a decrease in NK cells following radiotherapy in patients with breast cancer (32). In patients with non-metastatic NPC, we noted increased levels of CD16^+^CD56^+^ NK cells in the peripheral blood following CRT.

CRT is currently considered the primary and most effective treatment for NPC, which also impacts the immune function of patients. Whether the factors that predict the efficacy of CRT contribute to the optimal disease management of NPC has not been fully elucidated. Recent studies suggest that CRT plays a role in activating the tumor immune microenvironment, consequently impacting tumor response and prognosis (20, 33). Therefore, we hypothesize that circulating lymphocytes, as a crucial component of antitumor immunity, may influence the response to CRT. In the present study, a notable reduction in the percentage and absolute count of CD3^+^CD4^+^ T cells and CD19^+^ B cells was observed following completion of CRT. Conversely, a substantial increase in the percentage and absolute count of CD3^+^CD8^+^ T cells and CD16^+^CD56^+^ NK cells was noted one month after CRT. Furthermore, our study revealed that patients in CR exhibited significantly elevated baseline percentages and absolute counts of CD19^+^ B cells, along with higher percentages and absolute counts of CD3^+^CD4^+^ T cells at the end of CRT in comparison to patients in PR. Prior research has demonstrated a correlation between increased B cell levels and improved survival outcomes in patients with gastric and head and neck cancers (34, 35). The findings indicate that a significant proportion of non-metastatic NPC patients exhibited total T lymphocyte proportions within the normal range, with 37.4% of individuals demonstrating elevated CD8^+^ T cell subsets compared to typical values, suggesting a potential enhancement in immune function following one month of CRT. This provides a rationale for combining CRT with immune checkpoint inhibitors (ICIs). Furthermore, whether screening patients with normal or activated immune function to receive ICIs is more effective warrants further investigation.

This study has several limitations. We recognize that the retrospective assessment of lymphocyte subsets is constrained, and our inability to analyze lymphocyte levels at multiple post-CRT time points is primarily attributable to a limited sample size, potentially introducing calculation biases. Incorporating additional blood collection time points to detect antigen-specific T cells could yield a more precise characterization of the dynamic changes in circulating lymphocytes. The absence of longitudinal patient survival data hinders our ability to evaluate the prognostic significance of circulating lymphocyte subsets in NPC. Owing to the lack of adequate data, our study was unable to perform additional subgroup analyses for patients with PD and other variables. This restriction precluded the possibility of conducting more detailed subgroup analyses, which might have offered deeper insights into the specific characteristics and outcomes of various patient cohorts. Additionally, the unavailability of Epstein-Barr virus (EBV) DNA copy number data constrains our capacity to investigate the association between circulating lymphocyte subsets and EBV. Consequently, further prospective studies are imperative to substantiate our findings.

## Conclusions

Collectively, we comprehensively analyzed the dynamic changes in the percentages and absolute counts of circulating lymphocyte subsets in non-metastatic NPC patients undergoing standard CRT. The results indicated that CRT induced dynamic alterations in the composition of peripheral lymphocyte profiles. The baseline percentage and absolute count of CD19^+^ B cells, as well as the CRT-induced percentage and absolute count of CD3^+^CD4^+^ T cells, might serve as indicators to predict the clinical treatment response. These results provide additional evidence supporting the combination of CRT with immunotherapy in non-metastatic NPC patients and propose that circulating lymphocytes could potentially emerge as biomarkers for gauging treatment response. After validating our data in larger patient cohorts, the dynamic monitoring of lymphocyte subsets in the peripheral blood of non-metastatic NPC patients during treatment could offer opportunities for early assessment of treatment outcomes and may help stratify patients who are most likely to benefit from additional immunotherapy.

## Data Availability

The original contributions presented in the study are included in the article/**Supplementary Material**. Further inquiries can be directed to the corresponding author.

## Supplementary material

Supplementary Figure 1 Flow cytometry analysis of the expression of CD3 **(A)**, CD4 **(B)**, CD8 **(B)**, CD56 **(C)**, and CD19 **(C)** on peripheral blood cells before treatment, post-completion of CRT, and one month following CRT. T0 represented the values at the end of chemoradiotherapy; T1 represented the values at one month after chemoradiotherapy.
