# Supplementary figures and images for: Effect of chemoradiotherapy on the dynamics of circulating lymphocyte subsets in patients with non-metastatic nasopharyngeal carcinoma
Source: Front Oncol. 2025 Feb 12;15:1521836. doi: 10.3389/fonc.2025.1521836 (PMC11861370; doi:10.3389/fonc.2025.1521836)

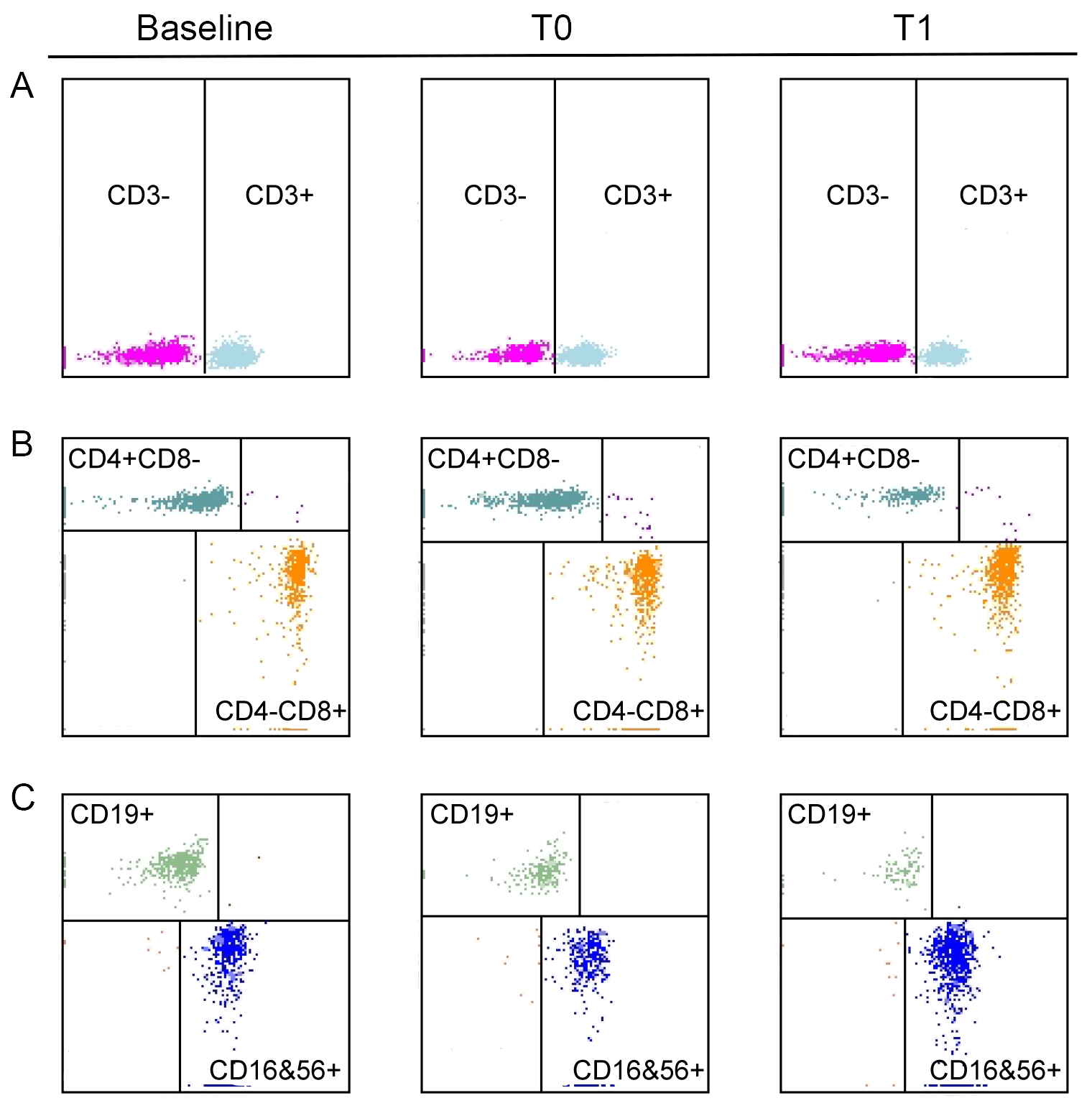

Supplement: Supplementary Figure 1 — Flow cytometry analysis of the expression of CD3 (A), CD4 (B), CD8 (B), CD56 (C), and CD19 (C) on peripheral blood cells before treatment, post-completion of CRT, and one month following CRT. T0 represented the values at the end of chemoradiotherapy; T1 represented the values at one month after chemoradiotherapy. [file Image1.tif]
